# Suggestions of Crown Work

**Published:** 1896-12

**Authors:** H. W. Allwine

**Affiliations:** Omaha, Neb


ARTICLE VI.

### SUGGESTIONS OF CROWN WORK.

BY H. W. ALLWINE, D. D. S., OMAHA, NEB,

To make sharp, well-formed cusps, by the Hollingsworth system, make the counter-die as directed. Smoke this, place it into the rubber ring and pour upon it Melotte's metal, nearly cold. Scrape from the die about the thickness of the gold to be swaged. Anneal the gold, partly swage, anneal again and drive home.

If the Hollingsworth cusps be not at hand, select a tooth having the size and cusps wanted. Imbed it in sand or any investment, exposing the cusps as desired. Place a ring about an inch in diameter, made of brass or any other metal, around this, and pour into the ring, over the cusps, some of Melotte's metal. This will give a counter-die. Make the die as directed above.

The die may be made first by taking an impression of the cusps in plaster and pouring the metal, quite cool, upon this, when the plaster has become hard and dry.

The metal should always be poured when it is just hot enough to flow.

To make an all gold bicuspid or molar crown, I proceed as follows: Prepare the side of the crown so a wire measure at the neck may be removed without breaking the wire. Then take a plaster impression of the tooth. Place around this a metal ring. Fill the impression of all the teeth, except the one wanted, with plaster. From the tooth wanted bevel the plaster to the upper edge of the ring. When this plaster is hard and dry, pour into it Melotte's metal. Now cut away the metal well up the side of the tooth, representing the cervical margin of the gum, so the band will pass well up. Dress the side of the crown so that the wire measure will pass over the metal tooth as it did over the natural one. This wire measure, cut and straightened, is the length of the gold required for the band. Fit the band down well all around and leave it long enough to extend just over the turn of the grinding surface. Scrape from the grinding surface of the metal tooth the thickness of the gold wanted here. Hammer and burnish the edge well down to the metal tooth and file it to a sharp edge. Now contour as desired. Place the band back of the metal tooth and with hammer and soft wood, drive a piece of well annealed gold to a perfect fit over the cusps and end of the band. Solder and dress down.

Any broken off molar or bicuspid may be thus capped if it be first built up with cement.—*Dental Digest.*

